# Experimental Study on the Influence of Ultraviolet Aging on the Shear Characteristics of HDPE Geomembrane/Sand Interface

**DOI:** 10.3390/polym18060776

**Published:** 2026-03-23

**Authors:** Hai Lin, Ruimin Chen, Haonan Li, Qiang Zhou, Guanghui Di, Xiaohaobo Wang

**Affiliations:** 1 School of Infrastructure Engineering, Nanchang University, 999 Xuefu Avenue, Honggutan New District, Nanchang 330031, China; linhai@ncu.edu.cn (H.L.); chenruimin@email.ncu.edu.cn (R.C.); lihaonan0603@163.com (H.L.); 71420@nchu.edu.cn (G.D.); 2 Engineering Research Center of Watershed Carbon Neutralization, Ministry of Education, Nanchang University, 999 Xuefu Avenue, Honggutan District, Nanchang 330031, China; 3 Tinhy Geosynthetics Co., Ltd., Diaozhen Industrial Zone, Zhangqiu District, Jinan 250204, China; haobo@tinhy.net

**Keywords:** geomembrane, ultraviolet aging, shear strength, interface, aging method

## Abstract

High-density polyethylene (HDPE) geomembranes (GMs) in landfill liners experience UV exposure during installation. While tensile strength deterioration after UV aging is known, changes in interfacial shear properties are rarely reported. This study investigates the evolution of interfacial shear behavior at the GM/sand interface by subjecting GM specimens to varying durations of indoor UV aging followed by direct shear tests. Underlying mechanisms were explored through tensile strength, melt flow index, crystallinity, and oxidation induction time (OIT) measurements. Results show that displacement required to reach peak shear strength for smooth geomembrane (GMS)/sand interface decreased with aging time (49.0–70.1% reduction), while no clear trend emerged for textured geomembrane (GMX)/sand interface. Following 80 days of UV exposure, the GMS/sand interfacial shear strength declined, with the peak friction angle dropping 20.6% from 26.2° to 20.8°. For the GMX/sand interface, the peak friction angle dropped to its lowest value of 31.2° after 40 days of exposure (from 34.3°), and then exhibited an increase with further UV aging. The large displacement shear strength followed a trend similar to that of the peak strength. Among the other tested indicators, the variation pattern of OIT with UV exposure exhibited the best correlation with the GMS/sand interface shear strength.

## 1. Introduction

Polymeric geosynthetic materials offer more reliable and sustainable alternatives to traditional techniques in geotechnical engineering. They have significantly advanced the development of ground improvement, slope stabilization, and environmentally resistant anti-seepage technologies [1,2]. High-density polyethylene (HDPE) geomembranes (GMs) are widely used in various engineering applications. These include reservoir watersheds, dams, landfills, expansive soil slopes, channels, oil wells, and mining operations. This widespread use is attributed to their low permeability, excellent environmental adaptability, strong deformation compatibility, and high cost-effectiveness [3,4,5,6,7]. In engineering applications, geomembranes are often positioned in direct contact with sand. The resultant interface serves as a crucial element of composite liner systems and is susceptible to shear slide under gravitational or external forces. During construction, geomembranes are often installed on-site and may remain exposed for extended periods. They are continuously subjected to UV radiation, temperature fluctuations, and oxidative influences [8,9]. Studies demonstrate that prolonged exposure to ultraviolet radiation degrades the tensile strength and elongation of geomembranes, in addition to other mechanical properties [10,11,12]. Rowe et al. [13] examined the material aging rate and service life of geomembranes based on their service environment. The service life is generally assessed by either the depletion of antioxidants or the reduction ratio of tensile strength due to aging. However, the factors determining the service life of geosynthetics in applications such as landfills depend not only on tensile strength. The change of interfacial shear characteristics between GM and sand during the aging process is crucially pertinent to the overall stability of the structure [14,15].

Extensive systematic tests have been performed on the interfacial shear properties between unaged geomembranes and soils [16,17,18], as well as on the evolution of the physicochemical properties of geomembranes in accelerated aging conditions [19,20,21]. Nevertheless, the specific influence of ultraviolet aging on the interfacial shear characteristics between geomembranes and sand has not been adequately examined. Currently, research on the shear properties of GM/sand interfaces has primarily focused on factors such as temperature, humidity, and surface roughness [22,23]. As a result, a relatively systematic understanding of their effects has been established. Ultraviolet aging, a common environmental element in construction, is inadequately studied in terms of its impact on interfacial shear performance [24]. Existing studies do not systematically cover different ultraviolet aging durations and normal stress levels. The continuous evolution of interfacial shear behavior with aging remains unestablished. Furthermore, the correlation between the microstructural evolution of the geomembrane and its interfacial shear behavior during aging remains unclear. Consequently, the underlying mechanism governing the degradation of interfacial shear characteristics is still poorly understood. The lack of relevant research not only leaves the durability design system for geosynthetics incomplete but also introduces uncertainty into the long-term safety of engineering projects. In recent years, research on the interfacial shear behavior of geosynthetics after aging has garnered increasing attention from the engineering community. The findings of these studies could provide new evidence for the long-term safety assessment of landfill systems [25,26]. Research in this field remains at a preliminary stage, and the relationship between the duration of ultraviolet aging and its impact on interfacial shear characteristics is inadequately comprehended. Furthermore, current standards and specifications lack explicit requirements for controlling the exposure time to ensure material durability.

To this end, this study performed accelerated indoor ultraviolet aging tests on geomembranes, utilizing graded aging durations ranging from 0 to 80 days. This study thoroughly examined the stress–displacement relationship, peak shear strength, and post-peak shear strength of the GM/sand interface using direct shear tests and microstructural analysis, considering different aging durations and normal stress levels. This study seeks to uncover the mechanism by which ultraviolet aging affects the interfacial shear behavior of GM/sand. It also aims to quantitatively assess the performance deterioration induced by aging. The findings are intended to provide a scientific foundation for durability design, exposure duration management, and long-term safety evaluation of geomembranes in ultraviolet-exposed environments.

## 2. Materials and Methodology

### 2.1. Materials

The geomembranes used in the tests were supplied by Tinhy Geosynthetics Co., Ltd. (Jinan, China) and consisted of the prevalent 1.5 mm thick smooth and textured high-density polyethylene (HDPE) geomembranes. The textured geomembrane featured a discrete, dotted rough surface produced using a spray-texturing method. The sand originated from Nanchang, Jiangxi Province, and represents a typical local sand with a dry density of 1.46 g/cm^3^. The specific mechanical properties of the geomembrane (GM) are listed in Table 1. The textured geomembrane (GMX) and the smooth geomembrane (GMS) utilized in this test were sourced from the identical production batch to guarantee the comparability of the results.

### 2.2. Ultraviolet Aging Test Method

**Figure 1:**
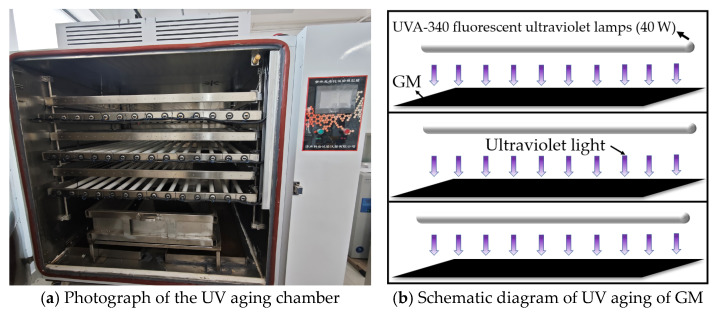
UV aging apparatus and method for GM.

To facilitate ultraviolet aging tests on extensive quantities of geosynthetic materials, a UVA-340 ultraviolet aging chamber (Figure 1a) was collaboratively constructed by Nanchang University and Jinan Koyan Co., Ltd. (Jinan, China). The UV accelerated aging experiments were conducted in accordance with the “Standard Practice for Operating Fluorescent Ultraviolet (UV) Lamp Apparatus for Exposure of Materials” (ASTM G154-23) [27]. The apparatus can support several layers of extensive geosynthetic specimens for simultaneous aging. The aging light source comprised UVA-340 fluorescent ultraviolet lamps (40 W), with an irradiance of 0.89 W/m^2^ as shown in Figure 1b. A cyclic aging regime was adopted: 8 h of UV irradiation at a black panel temperature of 60 °C, succeeded by 4 h of condensation at a black panel temperature of 50 °C. The cyclic alternation of UV irradiation and condensation was designed to simulate the diurnal cycle in natural environments. In this setup, the condensation phase corresponds to the dark period, replicating nocturnal conditions in outdoor settings. Geomembrane specimens were collected at 20-day intervals until the end of the 80-day aging period, yielding four sampling campaigns.

### 2.3. Interface Shear Test Method

To reflect the actual working conditions of bottom liners in modern sanitary landfills, this study adopted five normal stress levels: 50, 100, 200, 300, and 400 kPa. These levels were also chosen to maintain comparability with interfacial shear test results of geosynthetics obtained under standard laboratory conditions. Interfacial shear tests were performed using GM specimens that had been aged under indoor ultraviolet light for durations of 0, 20, 40, 60, and 80 days. A constant shear rate of 1 mm/min was applied in all tests to ensure measurement accuracy and maintain comparability with the findings of previous studies, such as that by Jones et al. [28].

**Figure 2:**
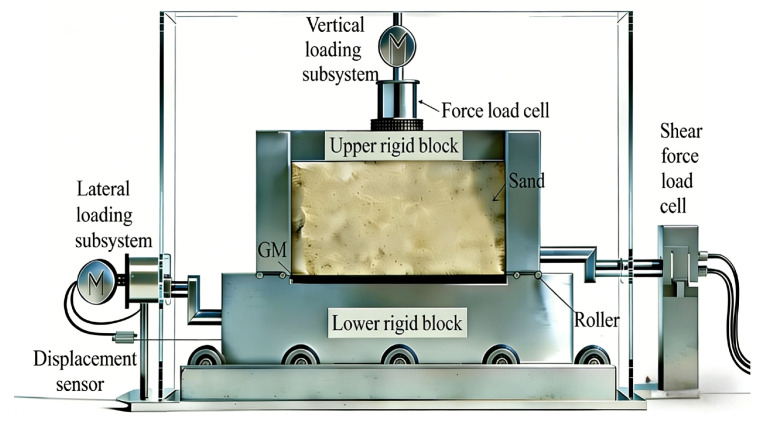
Schematic diagram of the stress–strain direct shear apparatus.

Shear tests on geosynthetic interfaces were conducted under the aforementioned conditions utilizing a stress–strain direct shear apparatus, a schematic diagram of which is shown in Figure 2. The procedures for interface shear testing are briefly described below. First, the GMX and GMS specimens that subjected to varying durations of UV aging were meticulously cut into rectangular shapes measuring 100 mm × 100 mm, ensuring the surfaces remained unscratched during the cutting procedure. Thereafter, a rigid spacer block of suitable height and the GM specimen were placed in the lower shear box to ensure that the specimen surface was level with the top of the lower box. Four roller pin rows were added on both sides of the lower shear box to diminish the frictional resistance between the upper and lower boxes. Subsequently, sand and the upper rigid block were systematically placed within the upper shear box. The upper and lower boxes were aligned and secured with screws. The assembled shear box was thereafter placed on the guide rail mechanism of the instrument’s rolling-bearing system, after which the instrument was closed to ensure seal integrity. Ultimately, normal stress was exerted and maintained until the settlement reached stabilization, after which the constant-rate shear test was initiated and proceeded until completion. Following the conclusion of the test, the upper and lower shear boxes were meticulously removed. The upper box, rigid top plate, and sand were removed in succession. The specimens were examined and photographed. The shear box was cleaned to prepare for forthcoming tests. The experimental data acquired by the computer were collected and processed to analyze the impact of UV aging on the shear characteristics. Research by Lin, H. et al. [3] on the interfacial shear characteristics of geosynthetics indirectly shows the good reliability of the apparatus and test method used in interface shear test, and confirms that the results are authentic and accurate.

### 2.4. Tensile Test Method

The tensile strength of geosynthetics is a key parameter for evaluating their mechanical performance. This study conducted tensile performance tests on the geomembranes in accordance with ASTM D6693-2004 [29]. A universal testing machine was used to evaluate the changes in the tensile strength at break, retention of tensile strength at break, and elongation retention rate of the HDPE membrane material throughout aging. The shape and dimensions of the specimens were based on the ASTM D638-22 [30], adopting dumbbell-shaped specimens of 25 mm × 115 mm, with a parallel section width of 6 mm and a length of 25 mm. In the strip tensile test, the specimen was clamped in the fixture and subjected to a tensile force until fracture, thereby allowing the measurement of its tensile strength and elongation. The testing machine employed delivered a consistent-rate tensile function. The fixture is designed to prevent specimen slippage during clamping, avoid damage to the specimen, and ensure the coplanarity of the gripping surfaces.

### 2.5. Microstructural Characterization Methods

#### 2.5.1. Differential Scanning Calorimetry (DSC)

This study utilized differential scanning calorimetry (DSC) to determine the melting point, crystallinity, and oxidation induction time (OIT) of the materials. All measurements were conducted using a DSC131EVO differential scanning calorimeter (DSC) manufactured by Setaram (Lyon, France). Before testing, the instrument was calibrated for both heat flow and temperature using an indium standard to ensure the accuracy of the measurement. The melting point and crystallinity were determined under a nitrogen atmosphere. Approximately 5 mg of the material was measured and placed in an aluminum crucible. A quantitative analysis of the relevant data was performed by monitoring the fluctuations in heat flow during the melting and crystallization processes of HDPE. Crystallinity was calculated based on the melting enthalpy data obtained from the crystallization process. Changes in the melting point can be used to evaluate the effect of aging on the polymer molecular structure. OIT determination was performed in strict accordance with ASTM D3895-19 [31]. The specimen was subjected to heating in high-temperature oxygen atmosphere, with continuous recording of temperature and heat flow. A distinct exothermic peak was observed upon oxidation of the sample. The OIT value is defined as the duration from the stabilization of the test temperature to the emergence of the exothermic peak. A higher OIT value signifies enhanced resistance against oxidative degradation of the HDPE geomembrane. A quantitative evaluation of the oxidation stability of the HDPE geomembrane can be conducted by comparing the OIT value with that of a standard or material with established performance.

#### 2.5.2. Melt Flow Rates (MFR) Test Method

The melt flow index (MFI) is a key parameter for characterizing the melt fluidity of thermoplastic polymers, such as HDPE geomembranes. It also serves as an indirect qualitative method for assessing polymer molecular weight. The operational procedure and foundational principles adhered to the standard ASTM D1238-23a [32], “Standard Test Method for Melt Flow Rates (MFR) of Thermoplastics by Extrusion Plastometer.” Prior to testing, the specimens were meticulously dried to exclude moisture, ensuring the precision of the test results. Initially, the specimen was fragmented into small granules during the experiment. Approximately 4–5 g of the HDPE geomembrane sample were weighed and placed into the barrel of the MFR tester. Prior to commencing the test, the specimen was preheated to the requisite temperature of 190 °C for polyethylene, and meticulous attention was given to eliminate any air bubbles within the barrel. After preheating, a standardized weight was placed atop the piston. The applied load then drives the polymer melt to extrude through a die of specified diameter. The test is conducted for 10 min. Upon completion, the mass of the extruded polymer was precisely measured. The MFI is quantified in grams of polymer extruded every 10 min (g/10 min).

## 3. Results

### 3.1. Shear Stress vs. Shear Displacement

**Figure 3:**
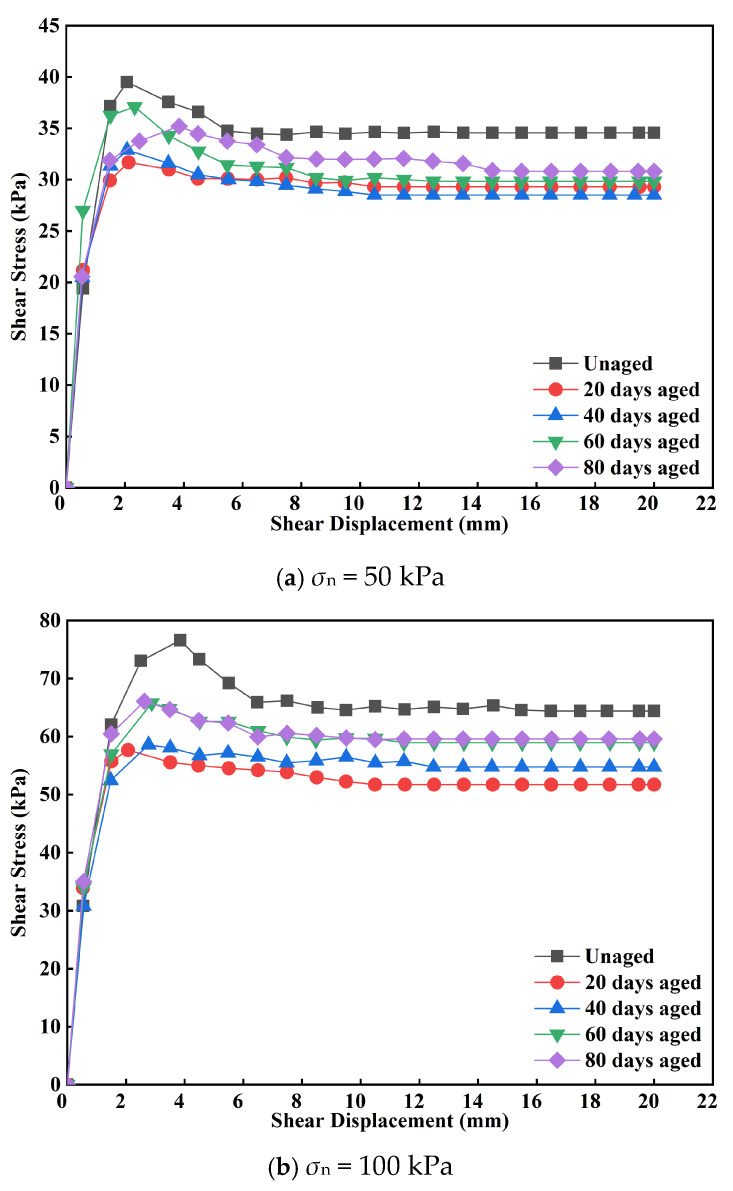

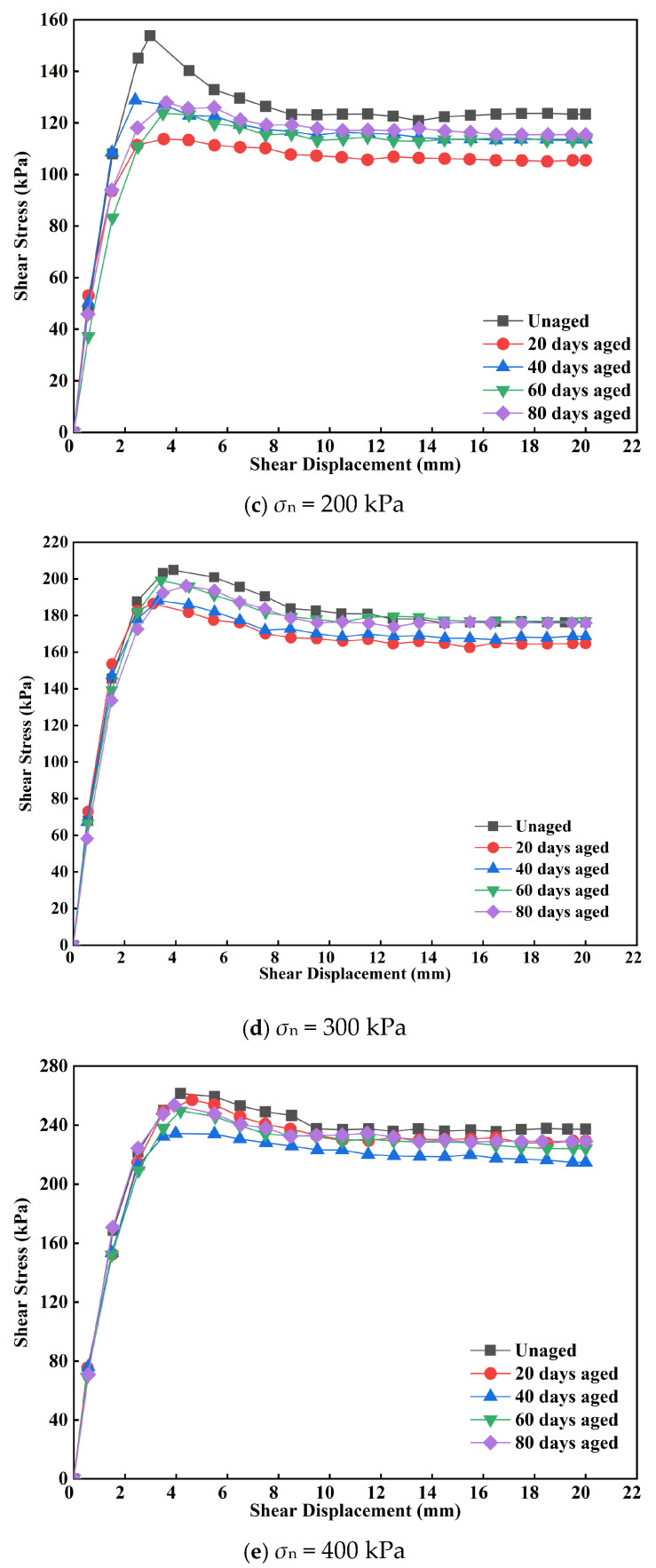
Shear stress vs. shear displacement for the GMX/sand interface subjected to the indoor UV aging for different durations.

**Figure 4:**
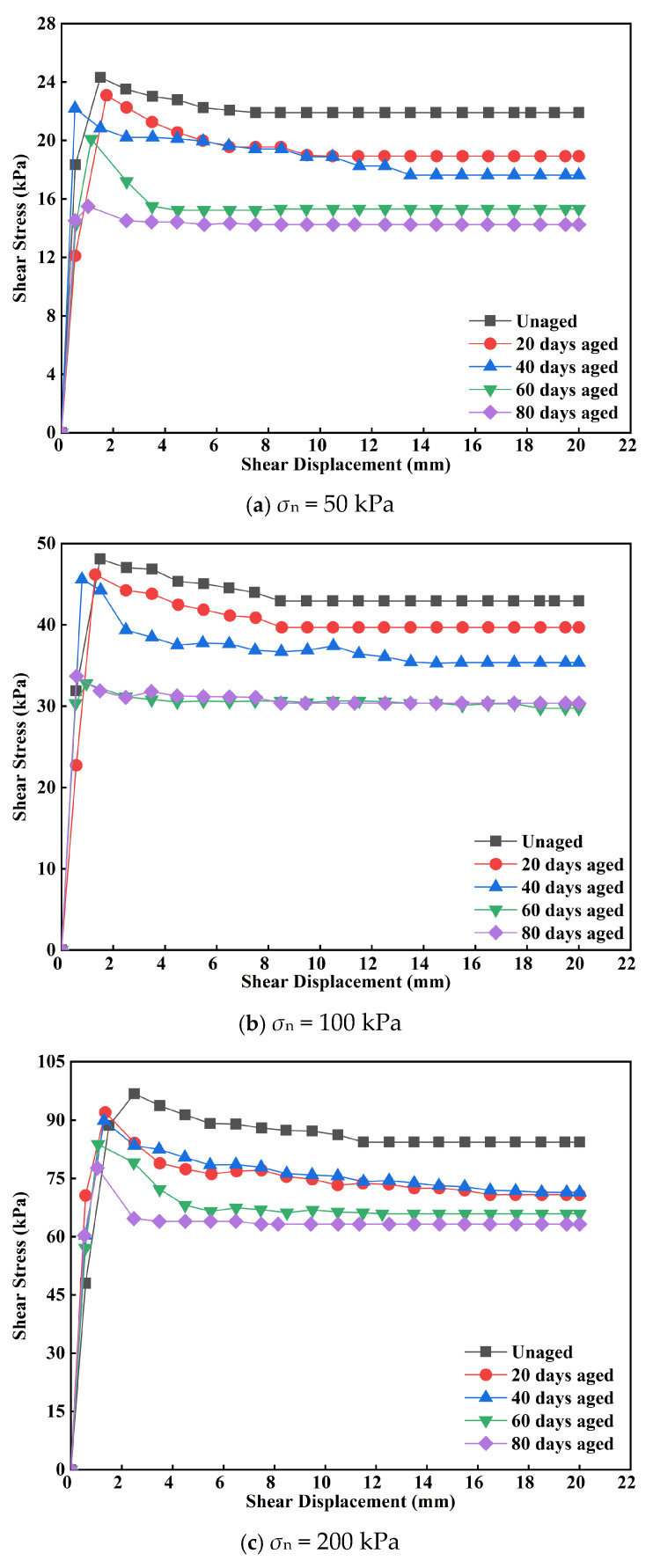

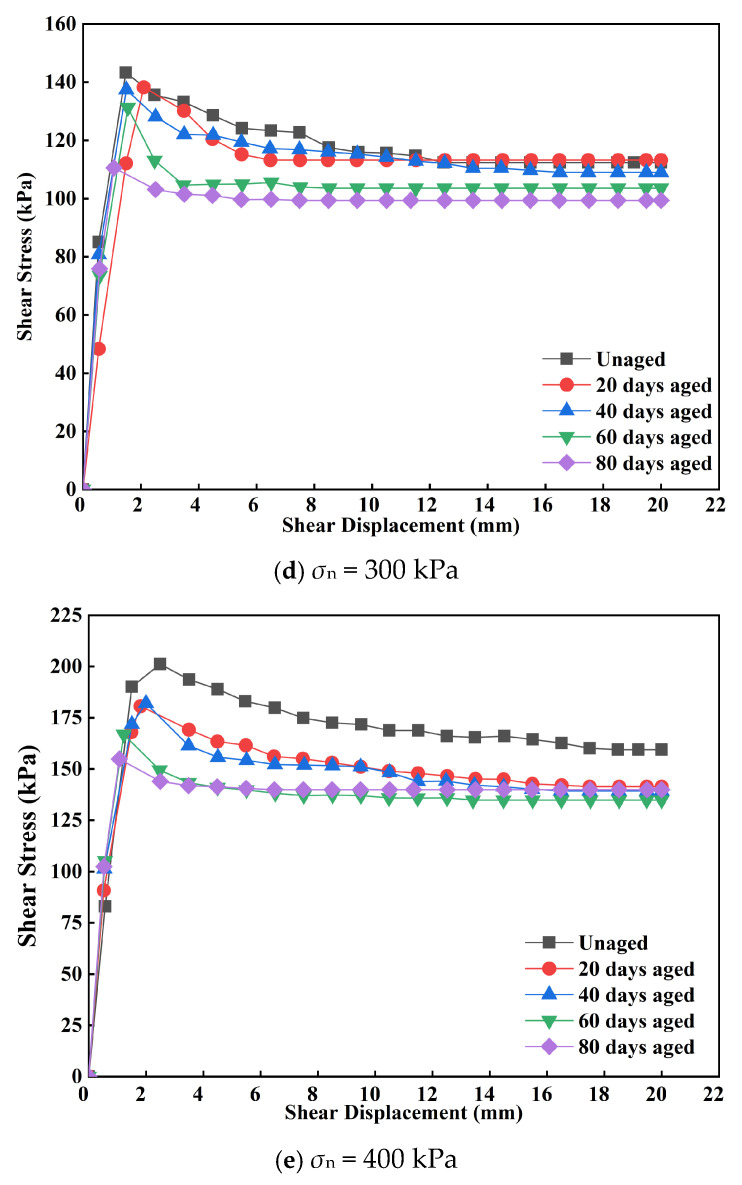
Shear stress vs. shear displacement for the GMS/sand interface subjected to the indoor UV aging for different durations.

The shear stress vs. shear displacement for the GM/sand interface subjected to the indoor UV aging for different durations are presented in Figure 3 and Figure 4. The peak shear displacement of the GMX/sand interface ranged between 2 mm and 5 mm. The shear stress stabilizes at a displacement of 7–10 mm. Under identical aging durations, both the degree of interface softening and peak shear displacement progressively increased with rising normal stress. At lower normal stress (σ_n_ = 50 and 100 kPa), the degree of softening first diminished, thereafter increasing with the duration of aging. At higher normal stress (σ_n_ = 200, 300 and 400 kPa), the fluctuation in the degree of softening with aging time was comparatively minor. Furthermore, the peak shear displacement increased with higher normal stress, although had no discernible relationship with aging duration.

For the aged GMS/sand interface, the shear stress peaked within a relatively small displacement range of 0.5–3 mm, then decreasing fast with additional displacement. As the displacement approached roughly 10–13 mm, the shear strength commenced stabilization. The peak shear displacement of this interface demonstrated an overall decline with prolonged aging, showing a drop of 49.0–70.1%. After 80 days of aging, the peak shear displacement was 0.58 mm under a normal stress of 100 kPa. At other stress levels, the peak shear displacement converged within a modest range of roughly 1.07 mm, indicating that UV aging considerably diminished the shear displacement of the GMS/sand interface.

### 3.2. Peak Shear Strength

**Figure 5:**
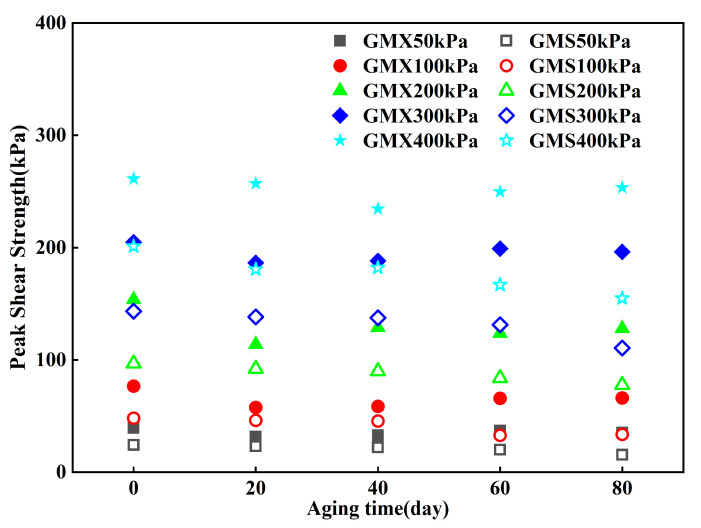
Relationship between peak shear strength of the indoor UV-aged GM/sand interface and aging duration.

The relationship between peak shear strength of the indoor UV-aged GM/sand interface and aging duration is shown in Figure 5. The GMX/sand and GMS/sand interfaces demonstrated different patterns in the variation of their peak shear strength. The peak shear strength of the GMX/sand interface first decreased and then increased with prolonged aging time, reaching its lowest value at t = 20 or 40 d. Compared with the unaged condition, the reductions in peak shear strength were 2–26% and 8–23%, respectively. Despite a minor resurgence in peak shear strength during the latter stages, the total strength remained inferior to that of the unaged condition. The decrease in strength of this interface was especially significant under normal stress of 100 and 200 kPa. In contrast, the peak shear strength of the GMS/sand interface showed a consistent decline with prolonged ultraviolet aging. This decline followed a staged degradation pattern. It decreased by 4–10% during the first 20 days, followed by a slower reduction of 0–4% between 20 and 40 days. After 40 days, the reduction rate accelerated again, reaching 4–27%. This pattern reflects the gradual decline of the interfacial shear performance with increasing aging duration.

**Figure 6:**
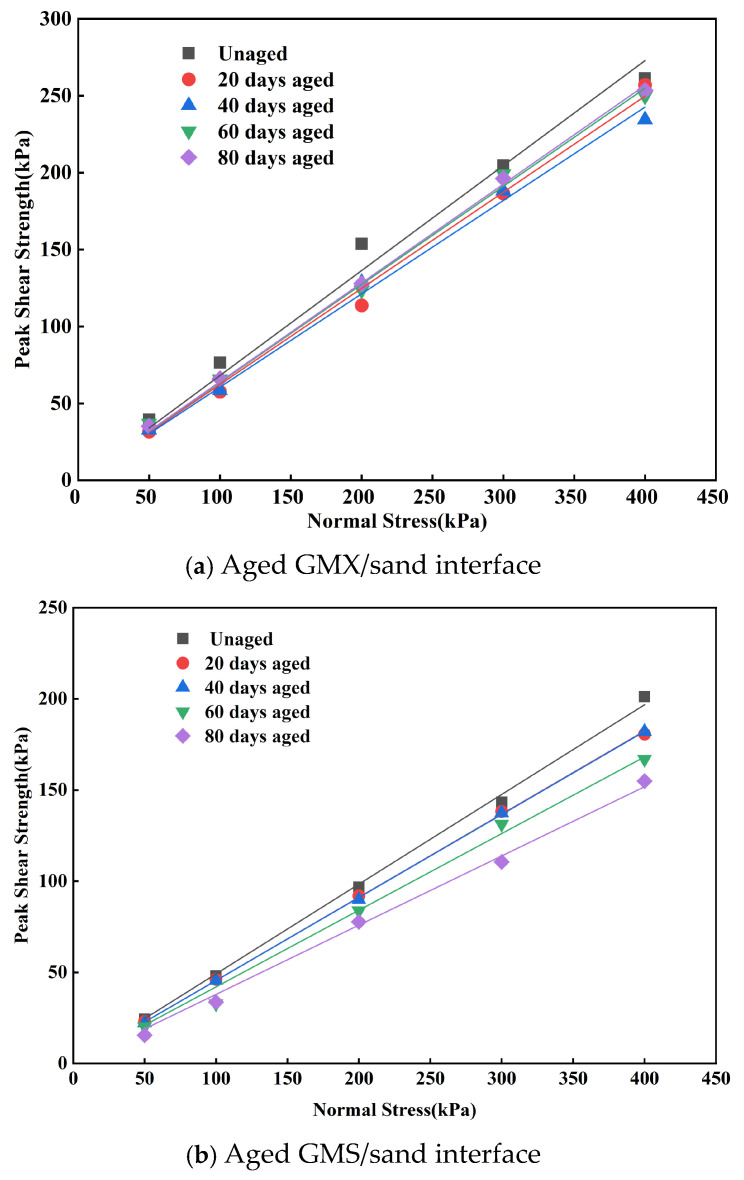
Peak shear strength envelopes of the GM/sand interfaces subjected to the indoor UV aging for different durations.

The peak shear strength envelopes of the GM/sand interfaces subjected to indoor UV aging for different durations are shown in Figure 6. The GMX/sand and GMS/sand interfaces exhibited a good linear correlation between peak shear strength and normal stress. Due to the very low peak cohesion values of both interfaces (GMX ≤ 7.52 kPa, GMS ≤ 4.87 kPa) and the absence of significant fluctuation with aging time, a cohesion-zero Mohr–Coulomb model was employed for fitting. This allowed the effect of UV aging on the interfacial shear characteristics to be reflected in the secant friction angle.

The peak shear strength envelope of the GMX/sand interface first decreased and then increased with aging time. At t = 40 d, the envelope reached its lowest position, corresponding to a decrease in the peak friction angle from the initial value of 34.3° to 31.2°, a reduction of approximately 9.0%. Subsequently, at t = 60 and 80 days, both the peak shear strength envelope and the friction angle showed a slight recovery, although they remained lower than those in the unaged state. In contrast, the strength envelope of the GMS/sand interface exhibited a continuous decline with aging time, with no indication of recovery. The peak friction angle decreased cumulatively from 26.2° to 20.8° after 80 days, yielding a total reduction of 5.4°. At aging durations of 20 and 40 days, the peak shear strength envelopes were nearly identical. Beyond 40 d, the rate of decrease in the peak friction angle accelerated. After 40 days, the rate of decline in the peak friction angle accelerated. This suggests that the impact of aging duration on the peak friction angle of this interface was relatively minor throughout the 20–40 days period of UV aging, but the aging effect of the UV source became much more relevant after 40 days.

### 3.3. Post-Peak Shear Strength

**Figure 7:**
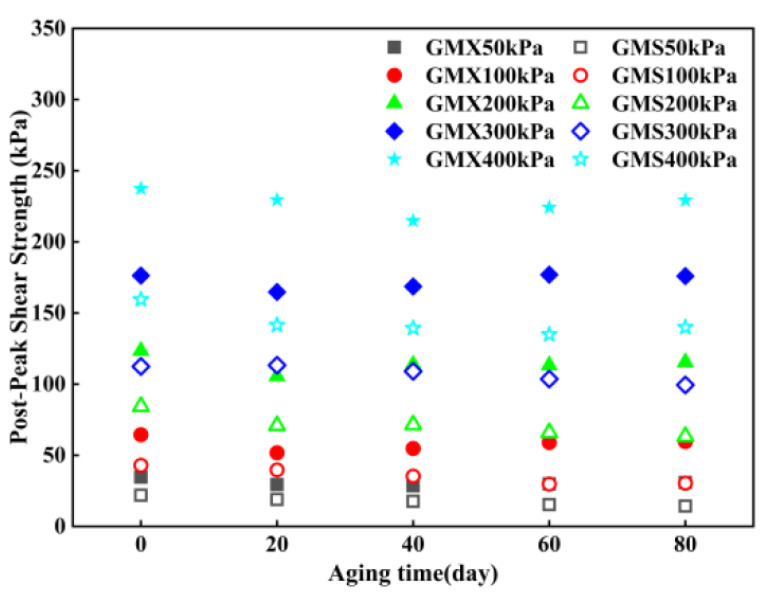
Relationship between post-peak shear strength of the indoor UV-aged GM/sand interface and aging duration.

The relationship between post-peak shear strength of the indoor UV-aged GM/sand interface and aging duration is shown in Figure 7. Subsequent to UV aging, the post-peak shear strength of the GMX/sand interface initially diminished and subsequently augmented. It diminished by 3–20% relative to the unaged condition over the initial 0–20 days, representing the primary degradation stage. Subsequently, the strength recovered by 0–7%, but the recovery rate gradually decelerated. The post-peak friction angle of this interface exhibited a similar trend, decreasing from 30.9° in the unaged state to 28.8° after 40 d of aging. Although it slightly recovered thereafter, it remained lower than its initial value. The post-peak shear strength of the GMS/sand interface exhibited a consistent decline, with a significantly increased rate of reduction during the 40–80 d period. The post-peak friction angle progressively decreased from an initial value of 21.6° to a stable value of 18.6° at 60–80 days of aging, yielding a total reduction of 3.0°. This indicates that the effect of UV aging on the interfacial shear characteristics tended to stabilize during this period.

**Figure 8:**
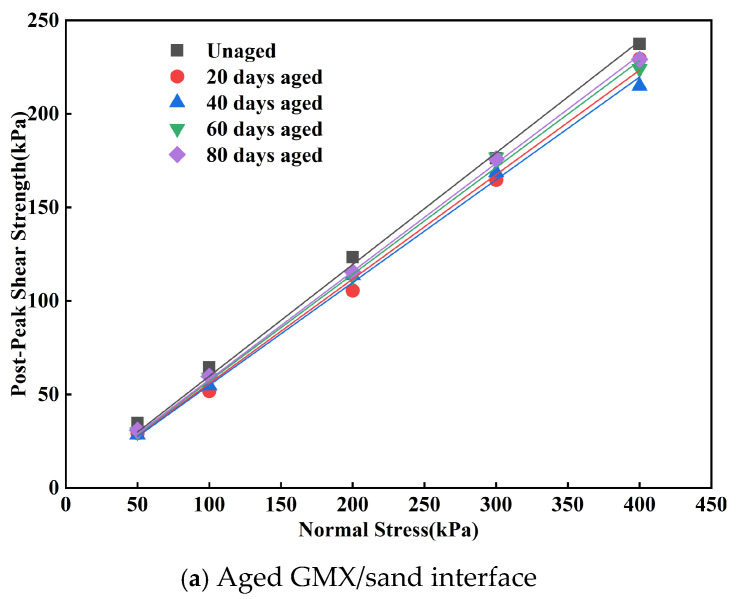

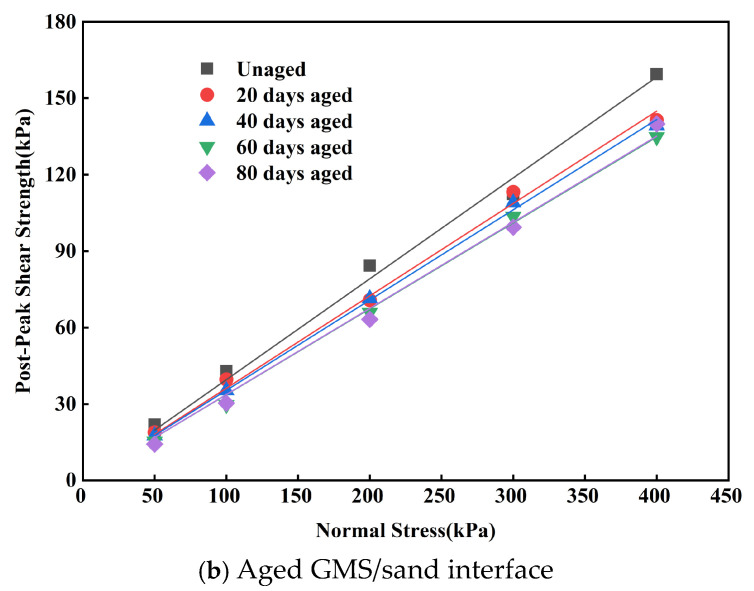
Post-peak shear strength envelopes of the GM/sand interface subjected to indoor UV aging for different durations.

Post-peak shear strength envelopes of the GM/sand interface subjected to indoor UV aging for different durations are presented in Figure 8. After UV aging, a significant linear correlation existed between the post-peak shear strength and normal stress for both the GMX/sand and GMS/sand interfaces, while the post-peak cohesion remained minimal. The trend in post-peak shear strength aligned with that of the corresponding peak shear strength, and the decrease in post-peak shear strength was typically less than that in peak strength. The results demonstrate that UV aging can diminish the extent of interfacial softening and impair interfacial shear characteristics. The shear failure morphology of the geomembrane specimens was examined. Under indoor ultraviolet aging conditions, the GMX specimens displayed no visible signs of surface damage. In contrast, all GMS specimens exhibited only minimal surface modifications.

## 4. Mechanism Analysis

### 4.1. Correlation Between Tensile Strength and Interfacial Shear Behavior Induced by UV Aging

**Figure 9:**
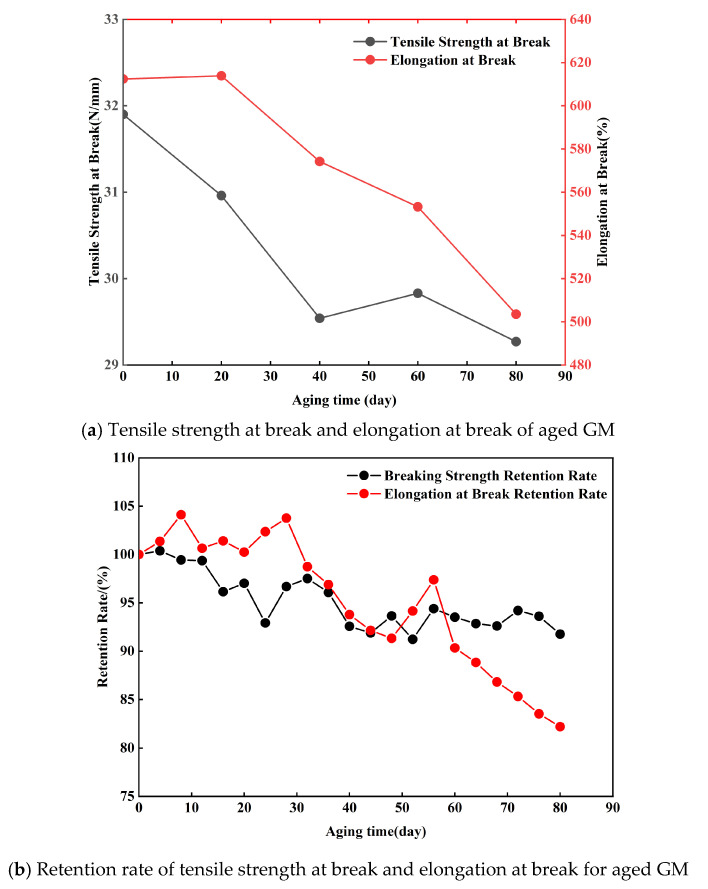
Variation in tensile mechanical properties of GM subjected to indoor UV aging.

Tensile tests were conducted on aged geomembrane (GM) specimens to examine the variation in their tensile properties with respect to indoor UV aging duration. Figure 9a illustrates the variations in tensile strength at break and elongation at break of the GM specimens subjected to varying durations of indoor UV aging. Figure 9b illustrates the corresponding retention rates of tensile strength at break and elongation at break. Figure 9b shows that under indoor UV aging, the retention rates of both tensile strength at break and elongation at break for the GM specimens generally exhibited a decreasing trend with increasing aging time. Following 80 days of indoor UV aging, the tensile strength at break of the GM specimens diminished by approximately 7%, while the retention rate of elongation at break reduced by almost 18%. Ultraviolet aging had a more pronounced effect on the elongation at break of the material. It caused a reduction in plastic deformation capacity and led to slight embrittlement. In contrast, its influence on the tensile strength at break was relatively minor. The cumulative effect of UV radiation affects the photo-oxidative aging performance of HDPE geomembranes, and prolonged exposure to UV light alters their chemical structure and physical properties. This occurs because, during the photo-oxidation process, UV light energy is absorbed by carbon–carbon double bonds in the polyethylene chains, instigating the generation of radicals. These radicals can further induce chain scission or crosslinking processes, resulting in a deterioration of the material’s mechanical characteristics. The extent of aging of the HDPE geomembrane did not increase proportionally with prolonged UV exposure time. This outcome can be ascribed to the existence of antioxidants or UV stabilizers within the substance. These additives operate by absorbing a portion of UV energy or neutralizing the radicals produced during the photo-oxidation process, therefore decelerating the material’s aging rate. As shown in Figure 9b, the tensile strength of the GM specimen aged for 60 d was slightly higher than that of the specimens aged for 40 and 80 d. The fluctuating variation observed in the mechanical properties of the aged specimens reflects two factors. It indicates non-uniformity in the irradiation process, as well as the influence of uncertainties in the sampling procedure on the test results.

The analysis combined with the interfacial shear test results indicates that UV aging led to a decrease in the elongation at break of the GM, a reduction in its plastic deformation capacity, and an increase in its surface smoothness. These changes collectively resulted in a weakening of the frictional interaction at the GM/sand interface, and this effect was more pronounced for the GMS/sand interface than for the GMX/sand interface. Therefore, with an extended aging time, the shear strength of the UV-aged GMS/sand interface showed a gradual decreasing trend, and the peak shear strength decreased correspondingly. The reduction in strength was greater for the GMS interface than for the GMX interface.

### 4.2. Correlation Between Microstructural Characteristics and Interfacial Shear Behavior Induced by UV Aging

**Figure 10:**
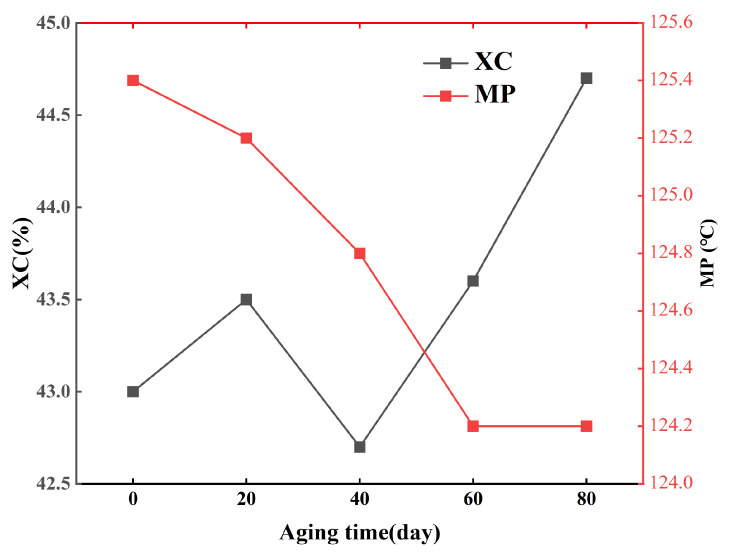
Variation of crystallinity and melting point of GM with aging time under indoor UV aging.

Differential scanning calorimetry (DSC) was employed to measure the crystallinity and melting point of the geomembrane after indoor UV aging at different time intervals. The results are shown in Figure 10. The figure illustrates that both the melting point and crystallinity of GM exhibited minimal variation with aging time under indoor UV light exposure. During accelerated UV aging tests, the melting point of the geomembrane remained at approximately 124 °C without significant change, while its crystallinity was maintained at a relatively stable level of approximately 44%. This can likely be attributed to two factors. First, HDPE possesses a high initial crystallinity and strong chemical stability; its long-chain carbon structure tends to remain stable under UV irradiation. Second, anti-aging agents, such as antioxidants and UV stabilizers, are likely to be added to HDPE. These additives are capable of absorbing or reflecting UV radiation. This action helps retard the photo-oxidation process. The molecular chains and crystalline regions are protected, contributing to the stability of both the melting point and crystallinity during aging. Thus, alterations in the melting point and crystallinity of the material were not the principal factors influencing the degradation of the shear characteristics of the GM/sand interface.

**Figure 11:**
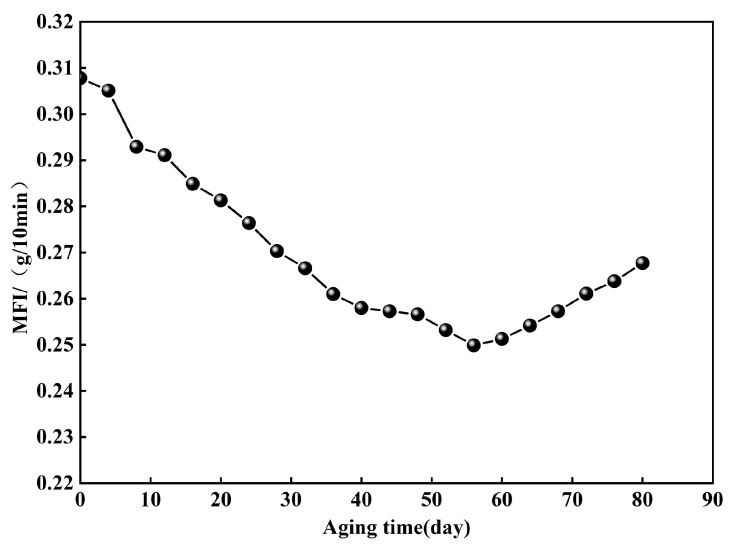
Variation of the MFI of GM with aging time under indoor UV aging.

In accordance with ASTM D1238-23a [32], the melt flow rate (MFR) of the geomembrane was assessed utilizing MFI tester. The variation in MFI of the HDPE geomembrane after UV aging is shown in Figure 11. Under the conditions of UV radiation, the MFI of the HDPE geomembrane decreased during the initial 50 days, indicating molecular crosslinking and a corresponding increase in molecular weight. An upward trend in the melt flow index was recorded over the subsequent 30 days. This observation indicates that, following the initial cross-linking stage, the HDPE geomembrane entered a transitional phase characterized by simultaneous cross-linking and molecular chain degradation. Over time, the degradation reaction induced by ultraviolet radiation became dominant. This led to the scission of molecular chains, a gradual decrease in molecular weight, and a subsequent increase in the MFI. This reduction in molecular weight over time was reflected in the macroscopic mechanical properties of the high-density polyethylene as a trend of deterioration. The test findings illustrate the complexity of the molecular structure and mechanical properties of HDPE geomembranes under accelerated UV aging. Crosslinking and degradation reactions dynamically influence the performance of materials at different stages. Even in the initial stage of photo-oxidative aging, a degree of oxidative degradation occurred. This degradation weakened the mechanical properties of the geomembrane, including its strength, toughness, and ductility. With further aging time, the MFI showed a slight increase, indicating that molecular restructuring tended toward a steady state. The interfacial shear strength of GMX/sand displayed a comparable evolution pattern to that of the MFI.

### 4.3. Oxidation Induction Time (OIT) Testing

**Figure 12:**
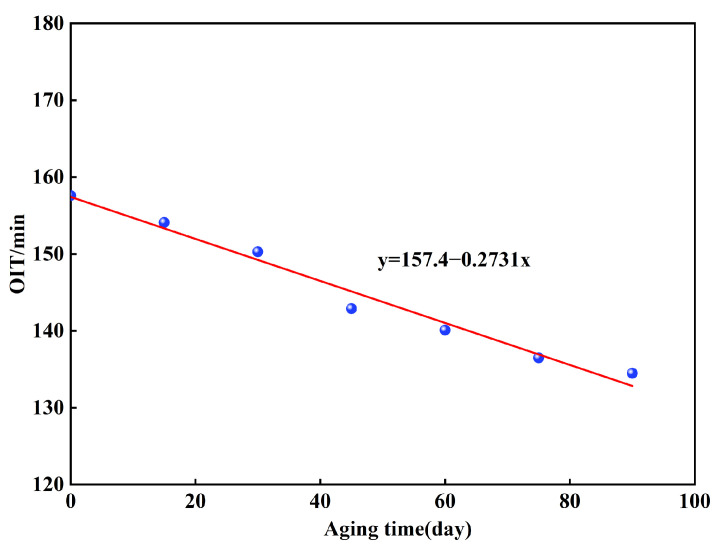
Effect of UV aging on the OIT of HDPE geomembranes.

The OIT test is a crucial method for assessing the oxidative stability of geomembranes. This test was performed to examine the rate of antioxidant consumption and variation in oxidation resistance of the geomembrane during long-term exposure to UV radiation. The change in the OIT values was expressed as a retention percentage, calculated using the following formula:

$${\mathrm{OIT}}_{r}\;=\;\frac{\mathrm{OIT}}{{\mathrm{OIT}}_{\mathrm{org}}}\;\times \;100\%$$

where ${\mathrm{OIT}}_{r}$ is the OIT retention percentage, OIT is the value measured for the aged sample at a specific aging time, and ${\mathrm{OIT}}_{\mathrm{org}}$ is the initial OIT value of the unaged sample. Figure 12 presents the fitted linear relationship between OIT and aging duration for the test specimens subjected to accelerated UV aging. Figure 12 shows that the OIT value consistently diminished as aging time increased, with a fitted linear slope k of −0.2731. After 90 days of accelerated UV aging, the OIT value decreased by approximately 14.66%, indicating that the geomembrane still retained sufficient antioxidants to ensure its long-term oxidative stability under field conditions. Owing to the presence of a photooxidation inhibitor in the geomembrane used in this experiment, the rate of free radical generation during the photooxidation reaction was slowed. The reactive free radicals generated during the photo-oxidation of the HDPE geomembrane were effectively scavenged by the antioxidant. This suppressed the progression of the photo-oxidation process into an auto-accelerating cyclic phase and prolonging the photo-oxidative lifespan of the material. Once HDPE materials have undergone a certain degree of aging under ultraviolet exposure, most of the antioxidants become depleted. As a result, the material’s resistance to photo-oxidative aging diminishes. This makes the photo-oxidative aging reaction more likely to enter an auto-accelerating stage. Based on the fitted linear equation for OIT, y = 157.4 − 0.2731x, the antioxidant depletion process under the accelerated UV aging conditions of this study was estimated to require approximately 576 days.

**Figure 13:**
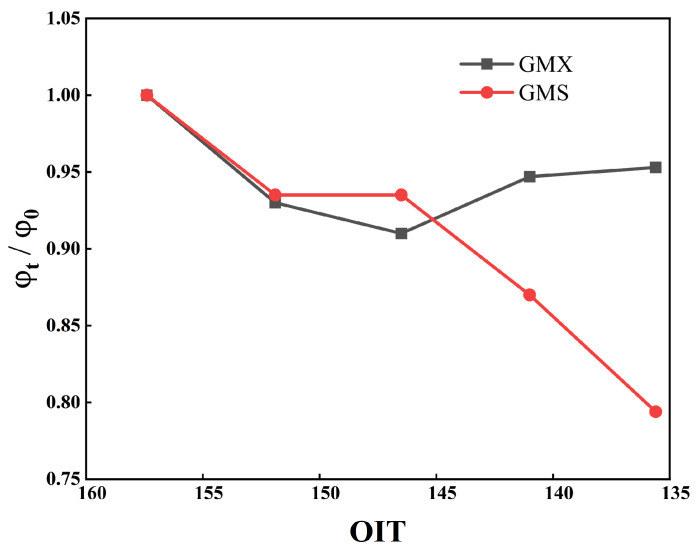
Relationship between the ratio of aged to unaged peak friction angles and the OIT.

The OIT values for aging times of t = 0, 20, 40, 60, and 80 days were computed using the fitted linear equation for OIT. Subsequently, the ratio of the peak friction angle at each aging time to that of the unaged state was plotted against the corresponding OIT value, as shown in Figure 13. As the aging time increased, the OIT value exhibited a continuous downward trend. The peak friction angle ratio of the GMX/sand interface exhibited a non-monotonic change, initially decreasing and then increasing. This indicates that the interfacial performance shows little correlation with antioxidant consumption. The partial recovery of interfacial shear strength arises from surface morphological changes induced by UV aging. In contrast, the peak friction angle ratio of the GMS/sand interface decreased continuously from 1.0 to 0.794, with a more pronounced reduction after 40 d of aging. This indicates that the interface is more sensitive to antioxidant consumption, and its frictional performance is more dependent on the integrity of the polymer chains, while its inherent resistance to aging damage is comparatively feeble.

## 5. Discussion

This study demonstrates that UV aging exerts distinctly different effects on the shear characteristics of the GMX/sand and GMS/sand interfaces. The peak shear strength of the GMS/sand interface exhibited a continuous decrease with aging time, whereas that of the GMX/sand interface first decreased and then increased (Figure 5 and Figure 6). The different evolutionary patterns of the shear characteristics between the two interfaces suggest that the degradation of interfacial shear performance is a complex process involving both bulk material deterioration and surface morphological evolution.

The crystallinity of the geomembrane exhibited an initial increase followed by a decrease during the early stage of UV aging (0–40 days), as shown in Figure 10, which was attributed to data scatter inherent in the experimental measurements. In the advanced stage of aging, a continuous increase in crystallinity was observed, potentially attributable to either post-crystallization of polymer molecules induced by UV irradiation [24] or to crosslinking reactions that increase the crystallinity of the GM polymer [8]. The crystallinity and melting point of the geomembrane following UV aging demonstrated maximum fluctuations of under 4%, maintaining considerable stability over the aging process. Concurrently, Anjana, R. K. et al. [8] reported that geomembranes are more susceptible to surface damage under UV aging. Accordingly, it can be inferred that ultraviolet aging exerts only a limited influence on the crystalline structure of the HDPE geomembrane matrix. The deterioration in geomembrane performance is instead primarily attributable to changes in surface characteristics. These surface changes arise from the dynamic evolution of chain crosslinking and scission occurring within the amorphous region.

In the preliminary phase of UV aging (0–40 days), the shear strength of both interfaces diminished. The reduction in MFI (Figure 11) indicated the occurrence of crosslinking reactions inside the molecular chains, resulting in an elevation of molecular weight. The decay in OIT (Figure 12) reflected the continuous consumption of antioxidants. Notably, the retention of elongation at break decreased by 18%, whereas the retention of tensile strength at break decreased by merely 7% (Figure 9b). This suggests that while crosslinking reactions may have partially compensated for the degradation of bulk strength, they also resulted in reduced surface ductility and increased embrittlement of the geomembrane [9,33]. The smooth surface of the GMS primarily relies on sliding contact between the geomembrane and sand particles for interfacial friction. The reduction in surface ductility thereby compromised the efficiency of frictional stress transfer, leading to a continuous decline in shear strength. The GMX/sand interface relies on both sliding friction and mechanical interlocking between its textured protrusions and sand particles. This dual mechanism reduces its dependence on the surface ductility of the geomembrane. Consequently, only a less reduction in shear strength was observed during the initial aging stage.

After 40 days of UV aging, the shear strength of the GMX/sand interface recovered while that of the GMS/sand interface continued to decline—a trend that presents an interesting contrast with the rebound of the MFI after 50 days (Figure 11). The increase in MFI indicates a deceleration of crosslinking reactions and the progressive dominance of chain scission. Despite chain scission often correlating with a decline in mechanical characteristics, the GMX interface demonstrated an anomalous increase in strength. This may be attributed to photo oxidation induced by UV radiation, which could have formed a slightly hardened layer or induced micro roughness on the textured protrusions of the GMX surface. It likely enhanced the mechanical interlocking with sand particles. This observation further substantiates that GMX possess superior shear resistance compared to smooth ones [34]. But this surface enhancement could not fully compensate for the progressive damage to the bulk material, resulting in limited recovery in strength, which remained below the unaged level.

While this study has revealed the evolutionary patterns of the shear characteristics of the GM/sand interface through indoor UV accelerated aging tests and has attempted to establish correlations between microscopic indicators and macroscopic behavior. Nevertheless, several limitations remain that merit further consideration. Inherent differences exist between the conditions employed in the UV accelerated aging tests and the actual service conditions encountered in landfill sites. The discrepancy makes it difficult to fully replicate the influence of complex environmental factors such as temperature and overburden pressure on the geomembrane aging process [11,13,21,24]. From a mechanistic perspective, a rough estimate was made of the time required for complete antioxidant depletion in the geomembrane under the indoor ultraviolet aging conditions used in this study. This estimate was based solely on the fitted linear OIT equation (y = 157.4 − 0.2731x). No prediction of the geomembrane’s service life under the condition was intended or performed. It should be noted that directly extrapolating service life from the linear OIT relationship warrants caution. Relying solely on the single indicator of OIT for service life estimation risks overlooking the influence of various other factors and is therefore inherently unrealistic [20,35].

## 6. Conclusions

This study investigated the influence mechanism of UV aging on the shear characteristics of the HDPE geomembrane/sand interface through 0–80 d graded UV accelerated aging tests, interface direct shear tests, and micro-scale property tests. The main conclusions are as follows:

(1) The peak friction angle of the GMS/sand interface progressively decreased from 26.2° to 20.8°, representing a reduction of 20.6%. Meanwhile, the peak shear displacement exhibited an overall decrease of 49.0–70.1%, converging to approximately 1.07 mm under all normal stress levels after 80 days of aging. The peak friction angle of the GMX/sand interface exhibited an initial decrease followed by a subsequent increase. It decreased from 34.3° to 31.2° at 40 days. Although a slight recovery was observed in the later stages, it remained below the unaged level.
(2) The progression of post-peak shear strength for both interfaces mirrored that of peak shear strength, with reductions in post-peak shear strength generally being less pronounced than those in peak strength. A robust linear correlation between interfacial shear strength and normal stress was consistently maintained, with cohesion remaining low and marginally influenced by aging.
(3) After 80 days of UV aging, the retention of elongation at break of the GM decreased by 18%, while the retention of tensile strength at break decreased by only 7%. MFI exhibited an initial decrease followed by an increase, reflecting the dynamic competition between crosslinking reactions and molecular chain scission. The variations in crystallinity and melting point of the GM were both less than 4%, indicating that UV aging has a limited effect on the crystalline structure.
(4) OIT exhibited a linear decrease with aging time and showed good correlation with the shear strength of the GMS/sand interface. The peak friction angle ratio of the GMS/sand interface decreased progressively with decreasing OIT, with a more pronounced reduction after 40 days of aging. In contrast, the peak friction angle ratio of the GMX/sand interface exhibited a non-monotonic trend, first decreasing and then increasing, and showed a weaker correlation with antioxidant consumption.

The study has investigated the influence mechanism of indoor UV aging on the shear characteristics of the GM/sand interface. Considering the differences between indoor UV aging and field natural aging, future work should conduct field exposure tests to establish the correlation between indoor UV aging and natural aging. This would help refine the durability design methodology for geomembranes and provide a scientific basis for optimizing the exposure duration requirements in relevant specifications.

## Figures and Tables

**Table 1:** Specific mechanical properties of the geomembrane.

Geosynthetics	GMX	GMS
Thickness, (mm)	1.5	1.5
Tensile strength at break, $\left(N\cdot {mm}^{-1}\right)$	31.6	50.8
Yield strength, $\left(N\cdot {mm}^{-1}\right)$	25.1	28.0
Elongation at break, (%)	782	855
Elongation at yield, (%)	14.0	13.0
Puncture resistance, (N)	573	646

## Data Availability

The original contributions presented in this study are included in the article. Further inquiries can be directed to the corresponding author.
